# Associations between lifestyle factors and levels of per- and polyfluoroalkyl substances (PFASs), phthalates and parabens in follicular fluid in women undergoing fertility treatment

**DOI:** 10.1038/s41370-023-00579-1

**Published:** 2023-07-22

**Authors:** Ida Hallberg, Richelle D. Björvang, Nermin Hadziosmanovic, Jacco Koekkoekk, Anne Pikki, Majorie van Duursen, Virissa Lenters, Ylva Sjunnesson, Jan Holte, Lars Berglund, Sara Persson, Matts Olovsson, Pauliina Damdimopoulou

**Affiliations:** 1 Department of Women´s and Children´s Health, Uppsala University, SE-751 85 Uppsala, Sweden; 2 Division of Obstetrics and Gynecology, Department of Clinical Science, Intervention and Technology, Karolinska Institutet, SE-141 86 Stockholm, Sweden; 3 Uppsala clinical Research Center, Uppsala University, SE-751 85 Uppsala, Sweden; 4 Environment and Health, Amsterdam Institute for Life and Environment, Vrije Universiteit Amsterdam, 1081 HV Amsterdam, The Netherlands; 5 Carl von Linnékliniken, SE-751 83 Uppsala, Sweden; 6 Julius Center for Health Sciences and Primary Care, University Medical Center Utrecht, Utrecht University, Utrecht, 3584 CG Utrecht, the Netherlands; 7 Department of Clinical Sciences, Swedish University of Agricultural Sciences, SE-750 07 Uppsala, Sweden; 8 School of Health and Welfare, Dalarna University, SE-791 88 Falun, Sweden; 9 Department of Public Health and Caring Sciences, Geriatrics, Uppsala University, SE-751 22 Uppsala, Sweden; 10 Department of Reproductive Medicine, Karolinska University Hospital Huddinge, SE-14186 Stockholm, Sweden

**Keywords:** Dietary exposure, Personal exposure, Phthalates, Endocrine disruptors, PFAS

## Abstract

**Background:**

Concerns have been raised whether exposure to endocrine-disrupting chemicals (EDCs) can alter reproductive functions and play a role in the aetiology of infertility in women. With increasing evidence of adverse effects, information on factors associated with exposure is necessary to form firm recommendations aiming at reducing exposure.

**Objective:**

Our aim was to identify associations between lifestyle factors including the home environment, use of personal care products (PCP), and dietary habits and concentrations of EDCs in ovarian follicular fluid.

**Methods:**

April-June 2016, 185 women undergoing ovum pick-up for in vitro fertilisation in Sweden were recruited. Correlation analyses were performed between self-reported lifestyle factors and concentration of EDCs analysed in follicular fluid. Habits related to cleaning, PCPs, and diet were assessed together with concentration of six per- and polyfluoroalkyl substances (PFASs) [PFHxS, PFOA, PFOS, PFNA, PFDA and PFUnDA], methyl paraben and eight phthalate metabolites [MECPP, MEHPP, MEOHP, MEHP, cxMinCH, cxMiNP, ohMiNP, MEP, MOHiBP]. Spearman’s partial correlations were adjusted for age, parity and BMI.

**Results:**

Significant associations were discovered between multiple lifestyle factors and concentrations of EDCs in ovarian follicular fluid. After correcting *p* values for multiple testing, frequent use of perfume was associated with MEP (correlation *ρ* = 0.41 (confidence interval 0.21–0.47), *p* < 0.001); hens’ egg consumption was positively associated with PFOS (*ρ* = 0.30 (0.15–0.43), *p* = 0.007) and PFUnDA (*ρ* = 0.27 (0.12–0.40), *p* = 0.036). White fish consumption was positively associated with PFUnDA (*ρ* = 0.34 (0.20–0.47), *p* < 0.001) and PFDA (*ρ* = 0.27 (0.13–0.41), *p* = 0.028). More correlations were discovered when considering the raw uncorrected *p* values. Altogether, our results suggest that multiple lifestyle variables affect chemical contamination of follicular fluid.

**Impact statement:**

This study shows how lifestyle factors correlate with the level of contamination in the ovary by both persistent and semi-persistent chemicals in women of reproductive age. Subsequently, these data can be used to form recommendations regarding lifestyle to mitigate possible negative health outcomes and fertility problems associated with chemical exposure, and to inform chemical policy decision making. Our study can also help form the basis for the design of larger observational and intervention studies to examine possible effects of lifestyle changes on exposure levels, and to unravel the complex interactions between biological factors, lifestyle and chemical exposures in more detail.

## Introduction

Involuntary childlessness is estimated to affect up to one in six couples in the European Union [1], with great social and financial consequences. Certain physiological factors are known to negatively affect reproductive health. For example, the chance of achieving a pregnancy is known to decrease with a woman’s increasing age and with a body-mass index (BMI) below or above the normal range. In addition to physiological factors in the male or the female, several lifestyle-factors are known to affect reproductive health. As an example, smoking negatively affect female fertility and reproductive health outcomes. Nevertheless, the reasons for infertility remain unknown even after thorough investigations in 10–20% of the couples [1].

The global decline in sperm count has received substantial attention among the general public, researchers and media [2, 3], and has become a well-established adverse health outcome associated with anti-androgenic endocrine disruptive chemicals (EDCs) in men. Fertility in women has, however, received less attention. Nevertheless, concerns have been raised about whether chemical exposure may alter reproductive functions and play a role in the aetiology of infertility also in women [4]. More specifically, we and others have shown that the level of exposure to plasticisers and persistent organic pollutants correlates with lower biomarkers of ovarian reserve, ovarian response to stimulation during in vitro fertilisation, longer time-to-pregnancy and higher odds for infertility [5–9]. These correlative cohort analyses have received causal support from experimental animal studies and led to the conclusion that fertility in women may be at risk due to EDC exposure [10, 11].

Depending on the chemical properties and differences in use, the possible routes of exposure differ between groups of chemicals. Phthalates, bisphenols and parabens are ubiquitously found in daily consumer-, personal care- and household products. Even though they are rapidly metabolised and excreted from the human body, exposure can be regarded as semi-persistent because the general population is continuously exposed. Consequently, measurable levels of parent compounds or metabolites are commonly found in human blood and urine [5, 12]. In addition, the compounds are also detected in follicular fluid, a liquid consisting of excretions from follicular cells and transudates from the circulation. This indicate direct exposure to the maturing oocytes in levels comparable or slightly below the levels measured in blood serum [5, 13]. Per- and polyfluoroalkyl substances (PFASs), on the other hand, are highly persistent compounds with half-lives in blood of years and with no metabolism of the compounds occurring [14]. This means that cumulative exposure over many years contributes to the body-burden of the chemicals, and a wide range of PFASs are found in human blood [15], with similar concentrations in ovarian follicular fluid [16]. The main routes of exposure to PFASs are considered to be through diet or water if regional contamination has occurred. In addition, the indoor environment and household dust as well as dermal contact also contribute to exposure [17–19].

Along with increasing evidence of adverse effects of EDCs on female reproductive function, knowledge on lifestyle factors associated with exposure is warranted to inform policies and form firm recommendations to women aiming for reducing exposure. We hypothesised that lifestyle factors are associated with EDC exposure in women contributing to levels present in the ovary. Therefore, the aim of the present study was to identify lifestyle factors that significantly correlate with concentrations of EDCs in ovarian follicular fluid.

## Methods

### Study population

We used a cohort of Swedish women who were undergoing ovum pick-up (OPU) for in vitro fertilisation (IVF) that has been described in our previous studies [5, 6, 16]. In brief, participants were recruited at the private Carl von Linné fertility clinic in Uppsala, Sweden, from April to June 2016. A total of 190 patients that underwent OPU for IVF during this period were asked to participate. Patients received written and oral information about the study. Patients who agreed to participate signed an informed consent form in accordance with the Declaration of Helsinki. Five patients declined and 185 patients agreed to participate (97% acceptance rate). Data were handled in compliance with relevant laws and institutional guidelines (the Swedish data protection law and the European General Data Protection Regulation). Biological samples were registered at Uppsala Biobank (IVO 627) following the Swedish law on biobanking in health care. Ethical permission was granted from the Regional Ethics Committee in Stockholm (Dnr 2015/798-31/2 with amendments 2016/360-32, 2019/2755-32).

### Questionnaire

Upon enrolment in the study, patients were asked to fill in a questionnaire about lifestyle and dietary habits, developed for the purpose of this study (available in the Supporting information, SI, Table S1). The questionnaire included questions on living environment, occupation, use of personal care products and diet as further described below. The questionnaire was pre-tested on a group of peers to clarify any sources of misinterpretations.

#### Personal data

Participants stated how long they had been living in Sweden (all their life, >5 years, <5 years or currently not living in Sweden). They also stated weather they used snuff (a popular tobacco product in Sweden) or if they, or any person in their household, was a smoker or had previously been a smoker.

#### Living environment

Cleaning habits were stated as the frequency of vacuum-cleaning or sweeping the floor in the household (daily, 2–3 times/week, once/week, twice/month, once/month, <once/month). Some chemicals in the home indoor environment accumulate in the dust. Various chemicals are released by electronics, textiles, ventilation pipelines, flooring materials (especially polyvinyl chloride, PVC), etc, and end up in the dust particles. How often the house is vacuum-cleaned and swept influences the levels of chemicals in housedust and exposure via inhalation and ingestion [18, 20, 21]. Therefore, we added these variables as a possible factor correlating with EDCs in follicular fluid. Participants further stated the type of flooring (wood, laminate, vinyl/PVC, carpet, cork, stone, linoleum) in the home by estimating the proportion of the house covered in each flooring type (<30%, 30–60%, >60%). Water supply to the household was recorded as municipal water or well water (bore water). Participants reported their use of odour-suppressants (air fresheners) in their home and whether they used plastic containers for microwave-heating of food.

#### Occupation

Main occupation for the last 6 months was categorised as employed/student/domestic work/unemployed/sick-leave or other. Current employment was specified as free text.

#### Personal care products (PCPs)

The participant scored their use of make-up, perfume, hair-spray, sun-screen, impregnated clothes (e.g., Gore-tex® material), and hair-dye as daily, weekly, monthly, seldom or never.

#### Diet

Dietary habits were assessed as how often (daily, weekly, monthly, seldom or never) products were currently consumed. Fish, specified as self-caught fish, fatty fish or white fish, and proportion of meat from hunted game was reported separately. Participants also stated whether they tended to choose an organic option of the products.

### Sample collection

The collection of ovarian follicular fluid has been described elsewhere [5, 6, 16]. In brief, participants underwent ovarian stimulation prior to OPU. Oocytes were aspirated through transvaginal ultrasound-guided ovarian follicular puncture according to standard procedures between 8–12 AM. After oocyte collection, all clear follicular fluid per participant was pooled, except for the first tube, which was always discarded to avoid potential contamination by flushing media used in the tubing system and plastic-associated chemicals. The sample was centrifuged at 500 × *g* to separate cells from the supernatant and subsequently stored at −80 °C together with blank samples that were prepared by pooling the flushing media (G-Rinse, Vitrolife, STOCKHOLM, SWEDEN) used for rinsing the tubing system (Wallace Single Lumen Oocyte Recovery System 17 G, CooperSurgical Fertility and Genomics, MÅLOV, DENMARK).

### Chemical analyses

Assessment of chemical concentrations in the follicular fluid samples has been described previously and details on performance characteristics are available in the SI, Table S2-S3 [5, 6]. In brief, prior to solid-phase extraction, parabens, bisphenols and phthalates were deconjugated. Target compounds were extracted on an Oasis MAX cartridge, washed and eluted with 2% FA in methanol. Chromatographic separation was carried out with an ultra-high performance liquid chromatography (ExionLC, Sciex, Foster City, CA, USA) coupled to a Turbo V electrospray ionisation source in negative mode prior to triple quadrupole mass selective detection (6500+, Sciex). For the quantification of PFASs, the LC-MS/MS as previously described by Lindh et al., 2012 [22] was used. In brief, samples were extracted using acetonitrile, glucoronidase and ammonium acetate buffer and spiked with isotopically labelled standards. Separation was carried out by liquid chromatography coupled with triple quadruple mass spectrometry (TRAP 5500, AB Sciex). Table S2-S3 in the SI contains the full list of isotopically labelled standards and list of chemicals, in total covering 10 bisphenols, 6 parabens, 16 phthalate metabolites and 8 legacy and emerging PFASs (Table S3, SI). In the current study, data on the Swedish samples were re-used to assess determinants of exposure. Chemical concentrations have been published elsewhere [5], and are available in Table S4, SI.

### Data pre-processing

Lifestyle factors were categorised or combined based on possible routes of exposure, and included living environment, recent use of personal care products (PCPs) and diet (Table 1). Factors that showed little or no variance within the study population (e.g., water-supply to the household, country of origin, dairy products) or were non-specific regarding time of exposure (e.g., dietary supplements, medication, hair-dye, sun-screen and odour-suppressants) were excluded from the analyses. Frequency of cleaning and type of flooring were included as variables contributing to the indoor environment. Flooring was categorised based on the proportion of the house with PVC-flooring, as PVC is known to contain high proportion of phthalates, and also based on the category used in the majority of flooring in the household. Use of plastic container for microwave heating of food was categorised as yes or no. Occupation was presented as six categories coded by IH based on common possible labour exposures [23, 24], (Table 1). The category Cosmetics included occupations such as hairdressers and stylists. Dental/hospital included occupations in environments with a high use of detergents and cleaning agents such as physicians, nurses, assistant nurses and dentists, but also cleaners. Manual workers such as machine operators or heavy traffic occupations was categorised as Industry. Occupations with much time spent outdoor such as child-care but also animal-husbandry were presented together as Outdoor. Managers and professionals working in an office environment were summed together as Office, this category also included two participants with related occupation that were currently on full-time maternity-leave. Clerical, service and sales workers were categorised as Service.

**Table 1:** Variables included as explaining factors from the questionnaire.

Variable	Details
Cleaning frequency	Frequency of vacuum-cleaning or sweeping of floor in household
	Scale: daily, 2–3 times/week, 1/week, 2/month, 1/month, <1/month
Flooring	Type of flooring: wood, laminate, vinyl/PVC, carpet, cork, stone, linoleum.
	Scale: proportion of the house covered in each type of flooring (<30%, 30–60%, >60%).
PVC_flooring	Proportion of house with PVC-flooring (none, <30%, 30–60%, >60%)
Diet	
- Meat	Frequency of eating habits^a^ Scale: weekly, monthly, seldom, never
- Fatty fish	
- White fish	
- Total fish	
- Hens’ egg	
Microwave use	Use of plastic containers to heat food in microwave
	Scale: yes, no
Personal care products	
- Make-up	Frequency of use of personal care products Scale: daily, weekly, monthly, rarely/never
- Perfume	
- Hairspray	
- ∑PCP	Average use of make-up, perfume and hairspray
-Impregnated clothing	

^a^Fish intake was also detailed as self-caught fish but due to low proportion of participants eating this category of fish frequently, this was excluded.

For the dietary habits, we included common protein sources, i.e., meat consumption, hens’ egg consumption and fish consumption. Fish consumption was categorised as white fish, fatty fish and mean of the two categories. Self-caught fish was excluded due to low frequency of consumption within the study population (three participants exceeding weekly consumption). Frequency of consumption was categorised into five categories (daily – weekly – monthly – seldom – never). Hen’s egg consumption also included a category for participants generally consuming eggs from organic production (yes/no). For PCP use, we included variables with frequent use, i.e, make-up, perfume, hairspray and impregnated clothes. In addition, a summation variable including products that could contribute to similar exposure was created by the average of use of make-up, perfume and hair-spray (∑PCP).

EDCs were included in the analyses if more than 90% of the follicular fluid samples exceeded level of detection (LOD). Consequently, 24 analytes were excluded and 22 of these had a LOD < 50% (for details, see Table S5). This gave a final dataset consisting of six PFASs (perfluoro-n-hexanesulfonate, PFHxS; perfluoro-n-octanoic acid, PFOA; perfluoro-octanesulfonate, PFOS; perfluoro-n-nonanoic acid, PFNA; perfluoro-n-decanoic acid, PFDA; and perfluoro-n-undecanoic acid, PFUnDA), one paraben (methyl paraben) and nine phthalate metabolites (mono-(2-ethyl-5-carboxypentyl) phthalate, MECPP; mono-(2-ethyl-5-hydroxyhexyl) phthalate, MEHPP; mono(2-ethyl-5-oxohexyl) phthalate, MEOHP; mono-(2-ethyl-1-hexyl) phthalate, MEHP; cyclohexane-1,2-dicarboxyl acid, mono-(7-carboxy-4- methylheptyl ester), cxMiNCH; mono-(4-methyl-7-carboxyheptyl) phthalate, cxMiNP; mono-(4-methyl-7-hydroxyoctyl)phthalate, ohMiNP; monoethyl phthalate, MEP; and mono(2-hydroxyisobutyl)phthalate, MOHiBP).

Spearman correlation between the four metabolites of di-2-ethylhexyl phthalate (DEHP) [MEHP, MECPP, MEHHP and MEOHP] was high (Figure S1, SI). Hence, a new variable based on their sum (∑DEHP) was created by summation of all metabolites. The primary metabolites of di-isononylphthalate (DiNP) are rapidly metabolised and not included in the panel of analytes. The two secondary metabolites, cxMiNP and ohMiNP, were measured and found to be highly correlated and a variable based on their molar sum (∑DiNP) was created.

Chemical quantification in sample blanks suggested minimal contamination for the included compounds (for details, see Table S6 in the SI).

### Statistical analysis

Descriptive statistics were presented as mean (standard deviation, SD) or number (percentage). The response rate was in general high. All lifestyle variables were analysed as ordinal scales as well as categorised as often (daily/weekly) vs seldom (less than weekly). The results were nearly identical and only the results from the ordinal scale analyses were therefore presented.

We used Spearman partial correlation to investigate the association between exposure to chemicals (PFHxS, PFOA, PFOS, PFNA, PFDA, PFUnDA, ∑DEHP, ∑DiNP, cxMiNCH, MEP, MOHiBP, methylparaben) and lifestyle factors. Kruskal-Wallis non-parametric test was used to test whether flooring material was related to chemical exposure. We used Spearman partial correlations with adjustment for the possible confounding variables age, parity and BMI identified through a directed acyclic graph available as Figure S2, SI. The Spearman partial correlation is equivalent to the Pearson correlation between the residuals of the linear regression of the ranks of the two variables on the ranks of the partialled variables. E.g., to calculate the partial correlation between perfume use and MEP the ranks of both variables were used as dependent variables in regression models with the ranks of age, parity and BMI as predictors, and the residuals from those models were correlated with the usual formula for Pearson correlation [25]. BMI can be influenced by some of the lifestyle factors in our study and may therefore be on a causal pathway to chemical exposure. However, BMI is also influenced by other factors (i.e., genetics) and as a conservative approach we made adjustment for BMI as well as age and parity in our analysis. We estimated a large number of correlation coefficients and to reduce the Type I error we calculated corrected p values. This correction may be made with the Bonferroni method which assumes tests to be independent and thus will be too conservative. With principal components analysis, which takes the dependence between tests into account, we estimated the effective number of independent tests (MEFF) [26] which was used to calculate the critical *p* value. *P* values were presented both as uncorrected raw *p* values, and as *p* values corrected for multiple testing. We also conducted sensitivity analyses to test robustness of findings by excluding MEHP and MEOHP from ∑DEHP. MEHP might be derived from unspecific hydrolysis of DEHP during sample collection. MEOHP is a secondary metabolite of DEHP not known to form from ex vivo hydrolysis. MEOHP was detected in some sample blanks at levels ~20% of levels found in follicular fluid. The correlation with the other metabolites, however, remained high (Figure S1). With the sensitivity analyses, the results were nearly identical and therefore the results from ∑DEHP including both MEOHP and MEHP were presented.

SAS (9.4) and R (i386 4.1.2) software was used for statistical analyses. Raw *p* values < 0.05 and corrected *p* values < 0.04/MEFF were considered statistically significant.

## Results

### Characteristics of study population

Descriptive statistics of the previously reported patient characteristics along with the lifestyle variables are presented in Table 2. As previously reported, the majority of the women were nulliparous with a median age of 35 (mean 34.7, standard deviation ±4.7, range 21–43) years at the time of recruitment [5, 6]. The cause of infertility was 29.0% female factor, 24.0% male factor, 7.5% both male and female factor and 39.5% unexplained infertility. The proportion of women that reported that they were currently smokers was 6%, and an additional 6% reported that they had previously been smokers to some extent. The majority (57.5%) reported that they consumed alcoholic beverages on weekly or monthly basis. Household flooring was dominated by wood or wood-tiles (61%) or a combination of several materials (18.5%) and few households were dominated by linoleum, laminate, carpet or PVC. Occupations in the category *office* were the dominating profession among participating participants (58.5%) followed by occupations in *hospital* environment (18.7%). The participants reported cleaning habits as every second week (53.5%) or less seldom (21%), while 24.5% reported cleaning on weekly or daily basis. Most participants ate hens’ eggs (78%) and meat (90%) on a regular basis, while the intake of white fish and fatty fish such was more variable (Table 2).

**Table 2:** Patient characteristics and lifestyle habits in the study population.

Variable	*N*	Mean (standard deviation, SD) or %
Age, mean (SD)	185	34.4 (4.7)
BMI, mean (SD)	184	23.5 (3.5)
Smoking, *n* (%)	185	
- Never		162 (87.6)
- Former/Current		23 (12.4)
Alcohol, *n* (%)	182	
- Daily/Weekly		43 (23.2)
- Monthly		63 (34.1)
- Rarely/Never		76 (41.1)
Cause of infertility, *n* (%)	185	
- Both male and female		14 (7.6)
- Female		54 (29.2)
- Male		44 (23.8)
- Unexplained		73 (39.5)
Duration of infertility, mean years (SD)	185	2.3 (1.6)
Parity, *n* (%)	185	
- 0		106 (57.3)
- ≥1		79 (42.7)
Occupation, *n* (%)	171	
- Cosmetics		3 (1.7)
- Dental/Hospital		32 (18.7)
- Industry		9 (5.3)
- Office		100 (58.5)
- Outdoor		18 (10.5)
- Service		9 (5.3)
Flooring, *n* (%)	185	
- Combination		34 (18.4)
- Laminate		18 (9.7)
- Linoleum		2 (1.1)
- Carpet		4 (2.2)
- PVC		14 (7.5)
- Wood/wood-tile		113 (61.1)
PVC-flooring, *n* (%)	185	
- None		131 (71.0)
- <30%		31 (16.8)
- 30–60%		18 (9.6)
- >60%		5 (2.6)
Cleaning, *n* (%)	181	
- Daily		4 (2.2)
- Weekly		40 (22.1)
- Every second week		99 (53.6)
- Monthly		34 (18.9)
- Seldom		4 (2.2)
Microwave use, *n* (%)	184	
- Does not use plastic containers		64 (34.7)
- Uses plastic containers		120 (65.3)
Diet, n daily/weekly (%), *n* seldom/never (%)		
- Fatty fish	179	87 (49%), 91 (51%)
- White fish	179	58 (32%), 120 (68%)
- Meat	183	164 (90%), 19 (10%)
- Hens’ egg	180	140 (78%), 40 (22%)
Personal care product use, *n* daily/weekly (%), *n* seldom/never (%)		
- Make-up	183	147 (80%), 36 (20%)
- Perfume	182	113 (78%), 69 (22%)
- Hairspray	173	51 (29%), 122 (71%)
- ∑PCP,^a^ mean (SD)	181	2.4 (0.7)
Other use, *n* daily/weekly (%), *n* seldom/never (%)		
- Impregnated clothes	174	25 (14%), 149 (86%)

^a^∑PCP created as the average use of make-up, perfume and hair-spray scaled from 1–4 where 1 indicate daily use and 4 never. Impregnated clothes were exemplified as Gore-tex® textile or shoes.

### Correlation between lifestyle factors and levels of chemicals in ovarian follicular fluid

We first assessed if typical co-variates of fertility, BMI, parity and age correlate with lifestyle factors. There were significant correlations between BMI and mean fish consumption (correlation *ρ* = 0.20, raw *p* value, p_raw_ = 0.004) and frequent use of perfume (*ρ* = −0.14, p_raw_ = 0.04). Links between parity and mean fish consumption (ρ =−0.15, p_raw_ = 0.03) and make-up use (*ρ* = −0.17, p_raw_ = 0.02) were also observed. Spearman’s correlation between lifestyle factors and age, BMI and parity can be found in Table S7.

**Fig. 1:**
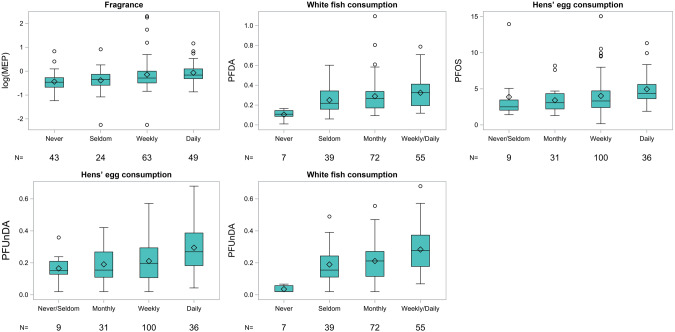
Concentration of chemicals in ovarian follicular fluid in women depending on lifestyle habits. Boxplots presenting the concentration of chemicals in each category of women. The line represents the median, the box is the interquartile range (IQR), whiskers are 1.5 × IQR, and dots represent extreme observed values > 1.5 × IQR. The trends were significant after adjusting for age, BMI and parity and correcting *p* values for multiple testing (p_cor_). MEP was associated with use of fragrance (correlation coefficient *ρ* = 0.34 (0.21 ; 0.47), p_cor_ = 0.0003). PFUnDA (*ρ* = 0.34 (0.20 ; 0.47), p_cor_ = 0.0005) and PFDA (*ρ* = 0.27 (0.13–0.41), p_cor_ = 0.03) were associated with white fish consumption. PFOS (*ρ* = 0.30 (0.15 ; 0.43), p_cor_ = 0.0069) and PFUnDA (*ρ* = 0.27 (0.12 ; 0.40), p_cor_ = 0.036) were associated with hen’s egg consumption.

**Fig. 2:**
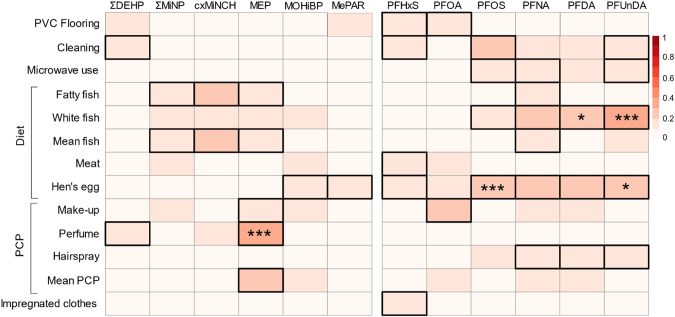
Correlation between chemical exposure and lifestyle factors resented as a heat-map where increasing correlation is depicted by increasing colour intensity. Thick black borders represent significant correlation adjusted for age, BMI, parity, and asterisks indicate correlation that remain significant after correcting *p* values for multiple testing. ***p < 0.001, *p < 0.05.

The correlations between lifestyle factors and levels of chemicals in ovarian follicular fluid adjusted for age, BMI and parity are presented in Table 3 (semi persistent chemicals) and Table 4 (persistent chemicals) and Figs. 1–2. The data could not be assumed to be normally distributed, therefore, Spearman’s correlation and the Kruskal-Wallis non-parametric test were used to investigate the association between exposure to chemicals and lifestyle factors. Correlations were adjusted for the possible confounding variables of age, parity and BMI (Figure S2, SI). The correlation without an adjustment for age, BMI and parity are available in the SI, Table S8. In general, the correlations remained the same with and without correction of *p* values for multiple testing and adjusting for BMI, age and parity; however, in some cases significance changed. This has been indicated where relevant.

**Table 3:** Correlation between lifestyle factors and levels of phthalate metabolites and metylparaben in ovarian follicular fluid adjusted for age, BMI and parity.

Phthalates, parabens		∑DEHP			∑DiNP			cxMiNCH			MEP			MOHiBP			Methylparaben		
	n	ρ (95% CI)	p-raw^a^	p-cor^b^	ρ (95% CI)	p-raw^a^	p-cor^b^	ρ (95% CI)	p-raw^a^	p-cor^b^	ρ (95% CI)	p-raw^a^	p-cor^b^	ρ (95% CI)	p-raw^a^	p-cor^b^	ρ (95% CI)	p-raw^a^	p-cor^b^
PVC-flooring	183	0.12 (−0.03;0.26)	0.11	ns	0.03 (−0.14;0.15)	0.96	ns	0.09 (−0.06;0.23)	0.27	ns	0.01 (−0.14;0.15)	0.95	ns	0.08 (−0.07;0.23)	0.27	ns	0.11 (−0.04;0.25)	0.15	ns
Cleaning	178	0.19 (0.05;0.33)	0.01	ns	0.07 (−0.08;0.21)	0.38	ns	0.06 (−0.09;0.21)	0.43	ns	0.05 (−0.10;0.19)	0.55	ns	0.05 (−0.10;0.20)	0.49	ns	0.04 (−0.11;0.19)	0.59	ns
Microwave use	183	0.06 (−0.09;0.20)	0.43	ns	0.06 (−0.09;0.20)	0.45	ns	0.10 (−0.05;0.24)	0.17	ns	0.09 (−0.06;0.24)	0.22	ns	0.02 (−0.13;0.16)	0.83	ns	0.01 (−0.14;0.15)	0.93	ns
Diet																			
Fatty fish	176	0.07 (−0.08;0.22)	0.36	ns	0.18 (0.03;0.32)	0.02	ns	0.26 (0.11;0.39)	0.0006	ns	0.15 (0.00;0.29)	0.05	ns	0.05 (−0.10;0.20)	0.48	ns	0.02 (−0.13;0.16)	0.84	ns
White fish	173	0.05 (−0.10;0.20)	0.53	ns	0.11 (−0.04;0.25)	0.16	ns	0.13 (−0.03;0.27)	0.10	ns	0.11 (−0.04;0.26)	0.15	ns	0.11 (−0.04;0.26)	0.15	ns	0.07 (−0.08;0.22)	0.36	ns
Mean fish	182	0.08 (−0.07;0.22)	0.31	ns	0.15 (0.00;0.29	0.05	ns	0.22 (0.08;0.36)	0.0028	ns	0.15 (0.01;0.29)	0.04	ns	0.03 (−0.12;0.17)	0.74	ns	0.02 (−0.13;0.17)	0.78	ns
Meat	181	0.08 (−0.06;0.23)	0.26	ns	0.15 (−0.00;0.29)	0.05	ns	0.05 (−0.10;0.19)	0.53	ns	0.02 (−0.13;0.16)	0.84	ns	0.12 (−0.03;0.26)	0.10	ns	0.08 (−0.07;0.23)	0.28	ns
Hens’ egg	177	0.06 (−0.09;0.20)	0.45	ns	0.02 (−0.13;0.17)	0.76	ns	0.08 (−0.07;0.23)	0.27	ns	0.09 (−0.06;0.24)	0.22	ns	0.17 (0.02;0.31)	0.03	ns	0.20 (0.05;0.34)	0.0091	ns
PCP^c^ use																			
Make-up	180	0.02 (−0.13;0.17)	0.78	ns	0.11 (−0.04;0.25)	0.15	ns	0.06 (−0.09;0.20)	0.43	ns	0.19 (0.04;0.32)	0.02	ns	0.11 (−0.04;0.25)	0.14	ns	0.03 (−0.12;0.18)	0.68	ns
Perfume	178	0.16 (0.01;0.30)	0.03	ns	0.03 (−0.12;0.18)	0.68	ns	0.11 (−0.04;0.26)	0.14	ns	0.34 (0.21;0.47)	<0.0001	0.0003	0.03 (−0.12;0.18)	0.66	ns	0.05 (−0.10;0.20)	0.48	ns
Hairspray	170	0.05 (−0.10;0.20)	0.50	ns	0.04 (−0.12;0.19)	0.63	ns	0.10 (−0.05;0.25)	0.14	ns	0.10 (−0.05;0.25)	0.21	ns	0.10 (−0.05;0.25)	0.18	ns	0.05 (−0.10;0.20)	0.49	ns
∑PCP	181	0.08 (−0.07;0.22)	0.29	ns	0.08 (−0.06;0.23)	0.27	ns	0.08 (−0.07;0.23)	0.28	ns	0.25 (0.10;0.38)	0.0009	ns	0.13 (−0.02;0.27)	0.08	ns	0.05 (−0.10;0.19)	0.53	ns
Impregnated clothes	171	0.00 (−0.15;0.15)	0.97	ns	0.00 (−0.15;0.15)	0.96	ns	0.01 (−0.15;0.16)	0.95	ns	0.02 (−0.13;0.18)	0.75	ns	0.00 (−0.15;0.15)	0.97	ns	0.01 (−0.15;0.16)	0.95	ns

^a^adjusted for Age, BMI and parity,^b^corrected for multiple testing using MEFF, ns indicate non significant values [22]. ^c^personal care product, ∑PCP the average use of make-up, hair-spray and perfume. ∑DEHP was created by summation of all four metabolites of di-2-ethylhexyl phthalate (DEHP) [mono-2-ethylhexyl phthalate (MEHP), mono-(2-ethyl-5-carboxypentyl) phthalate (MECPP), mono-(2-ethyl-5-hydroxyhexyl) phthalate (MEHHP), and mono(2-ethyl-5-oxohexyl) phthalate (MEOHP)] divided by their molecular weight. ∑DiNP was created by summation of the two secondary metabolites mono-(4-methyl-7-hydroxyoctyl)phthalate (cxMiNP) and monoethyl phthalate (ohMiNP) divided by their molecular weight.

**Table 4:** Correlation between lifestyle factors and levels of per- and polyfluoroalkyl substances (PFASs) in ovarian follicular fluid adjusted for age, BMI and parity.

PFASs		PFHxS			PFOA			PFOS			PFNA			PFDA			PFUnDA		
	n	ρ (95% CI)	p-raw^a^	p-cor^b^	ρ (95% CI)	p-raw^a^	p-cor^b^	ρ (95% CI)	p-raw^a^	p-cor^b^	ρ (95% CI)	p-raw^a^	p-cor^b^	ρ (95% CI)	p-raw^a^	p-cor^b^	ρ (95% CI)	p-raw^a^	p-cor^b^
PVC-flooring	181	0.15 (0.00;0.29)	0.04	ns	0.19 (0.05;0.33)	0.03	ns	0.05 (−0.10;0.19)	0.53	ns	0.06 (−0.09;0.20)	0.46	ns	0.03 (−0.12;0.17)	0.73	ns	0.00 (−0.15;0.15)	0.98	ns
Cleaning	176	0.19 (0.04;0.33)	0.01	ns	0.01 (−0.14;0.16)	0.89	ns	0.24 (0.09;0.38)	0.001	ns	0.14 (−0.01;0.28)	0.06	ns	0.11 (−0.04;0.25)	0.16	ns	0.16 (0.02;0.31)	0.03	ns
Microwave use	181	0.04 (−0.11;0.19)	0.59	ns	0.04 (−0.11;0.18)	0.73	ns	0.17 (0.02;0.31)	0.02	ns	0.16 (0.02;0.30)	0.03	ns	0.14 (−0.01;0.28)	0.07	ns	0.20 (0.06;0.34)	0.0064	ns
Diet																			
Fatty fish	174	0.00 (−0.15;0.15)	0.99	ns	0.05 (−0.10;0.20)	0.39	ns	0.06 (−0.09;0.21)	0.43	ns	0.15 (0.00;0.29)	0.05	ns	0.07 (−0.08;0.22)	0.34	ns	0.09 (−0.06;0.24)	0.24	ns
White fish	172	0.04 (−0.11;0.19)	0.63	ns	0.09 (−0.07;0.23)	0.26	ns	0.19 (0.04;0.33)	0.01	ns	0.25 (0.11;0.39)	0.0008	ns	0.27 (0.13;0.41)	0.0003	0.03	0.34 (0.20;0.47)	<0.0001	0.0005
Mean fish	179	0.00 (−0.15;0.15)	0.97	ns	0.02 (−0.13;0.17)	0.81	ns	0.06 (−0.08;0.21)	0.40	ns	0.15 (0.00;0.29)	0.05	ns	0.10 (−0.05;0.24)	0.18	ns	0.14 (−0.01;0.28)	0.06	ns
Meat	178	0.16 (0.01;0.30)	0.03	ns	0.13 (−0.01;0.28)	0.08	ns	0.02 (−0.13;0.17)	0.76	ns	0.08 (−0.07;0.22)	0.32	ns	0.01 (−0.14;0.16)	0.90	ns	0.03 (−0.12;0.17)	0.74	ns
Hens’ egg	175	0.16 (0.01;0.30)	0.0345	ns	0.13 (−0.02;0.27)	0.09	ns	0.30 (0.15;0.43)	<0.0001	0.0069	0.23 (0.09;0.37)	0.0021	ns	0.25 (0.11;0.39)	0.0007	ns	0.27 (0.12;0.40)	<0.0001	0.0005
PCP^c^ use																			
Make-up	178	0.03 (−0.11;0.18)	0.65	ns	0.24 (0.09;0.37)	0.0014	ns	0.05 (−0.10;0.20)	0.48	ns	0.13 (−0.01;0.28)	0.08	ns	0.13 (−0.02;0.27)	0.09	ns	0.09 (−0.06;0.24)	0.23	ns
Perfume	176	0.03 (−0.12;0.18)	0.71	ns	0.04 (−0.11;0.19)	0.61	ns	0.03 (−0.12;0.17)	0.74	ns	0.04 (−0.11;0.19)	0.62	ns	0.00 (−0.15;0.15)	0.96	ns	0.04 (−0.11;0.19)	0.60	ns
Hairspray	168	0.03 (−0.12;0.18)	0.85	ns	0.07 (−0.09;0.22)	0.38	ns	0.12 (−0.03;0.27)	0.13	ns	0.17 (0.01;0.31)	0.03	ns	0.20 (0.05;0.35)	0.0085	ns	0.19 (0.04;0.33)	0.015	ns
∑PCP	179	0.00 (10.15;0.15)	1.00	ns	0.12 (−0.02;0.27)	0.10	ns	0.05 (−0.10;0.20)	0.49	ns	0.14 (−0.01;0.28)	0.06	ns	0.11 (−0.03;0.26)	0.13	ns	0.08 (−0.07;0.23)	0.28	ns
Impregnated clothes	170	0.15 (0.00;0.30)	0.05	ns	0.02 (−0.13;0.17)	0.77	ns	0.04 (−0.12;0.19)	0.64	ns	0.03 (−0.12;0.19)	0.65	ns	0.05 (−0.10;0.20)	0.50	ns	0.09 (−0.06;0.24)	0.23	ns

^a^adjusted for Age, BMI and parity,^b^corrected for multiple testing using MEFF, ns indicate non significant values [22], ^c^personal care products, ∑PCP the average use of make-up, hair-spray and perfume.

MEP was positively associated with frequent use of perfumes (*ρ* = 0.34 (0.21; 0.47), p_raw_ < 0.0001, p_cor_ = 0.0003) (Table 3, Fig. 1). MEP was also associated with make-up and average PCP use, but these associations lost significance after correction of *p* values (Table 3). ∑DEHP was associated with frequent cleaning and perfume use, but these associations were not significant after correction of *p* values. cxMiNCH and ∑DiNP were positively associated with fish consumption and methylparaben correlated with hens’ egg consumption in both the adjusted and the non-adjusted analyses but lost significance after correction of *p* values. We did not observe any correlation between the semi-persistent chemicals and use of plastics in microwave heating of food or flooring material assessed as categories (*p* > 0.05) or as proportion of PVC flooring in the household (Table 3).

Hens’ egg consumption was associated with PFAS exposure; in particular PFOS (*ρ* = 0.30 (0.15; 0.43), p_raw_ < 0.0001, p_cor_ = 0.0069) and PFUnDA (*ρ* = 0.27 (0.12 ; 0.40), p_raw_ = 0.0004, p_cor_ = 0.036) showed significant associations (Table 4, Fig. 1). The association was not seen for PFUnDA after correction of *p* values in the unadjusted correlations (Table S7, SI). Participants choosing organic hen’s eggs for consumption had higher levels of PFUnDA (p_raw_ < 0.0001, p_corr_ = 0.01). They also had higher levels of PFOS, PFNA and PFDA, although the significance did not persist after correction of *p* values.

PFAS levels were also associated with fish consumption (fatty fish, white fish or average fish consumption); however, only PFUnDA (white fish, *ρ* = 0.34 (0.20 ; 0.47), p_raw_ < 0.0001, p_cor_ = 0.0005) and PFDA (white fish, *ρ* = 0.27 (0.13–0.41), p_raw_ = 0.0003, p_cor_ = 0.0275) showed significance after correcting *p* values for multiple testing (Table 4, Fig. 1). In the unadjusted analyses, association between fish consumption and PFNA was also significant (Table S7, SI). A lower frequency of cleaning was in general associated with higher PFAS-exposure, but this correlation lost significance after applying adjustment for multiple testing or adjusting the models for co-variates. Similarly, some associations were seen between PFAS exposure and microwave use, PCPs or impregnated clothes and PVC-flooring before correction of *p* values (Fig. 2).

We did not assess correlation between occupational categories and EDC concentrations in follicular fluid due to the limited number of participants in each category.

### Discussion

We present associations between lifestyle including dietary habits and PCP use with the levels of chemicals present in the ovarian follicular fluid of women of reproductive age seeking assisted reproductive technologies for conception. Using the same cohort of participants, we have previously shown that higher exposure to PFASs was associated with higher antral follicle count and ovarian response to gonadotropin stimulation but lower embryo quality [6]. Furthermore, DEHP, potentially PFUnDA and the overall mixture of the chemicals present in the follicular fluid were associated with lower ovarian sensitivity [5]. These observations suggest that chemical contamination in ovarian follicular fluid may have an impact on fertility, and it is therefore important to reduce the exposure. Our current study shows that exposure correlated with the lifestyle of the participating women. Chemical contamination of the follicular fluid is a direct measure of the exposure of the maturing oocyte. Therefore, this suggests that lifestyle choices could represent an actionable solution for reducing exposure of oocytes, especially for the non-persistent chemicals, and potentially improve treatment outcomes in women undergoing assisted reproduction and in women in general.

The study population represented the average population of Swedish women of similar age in regard to BMI and smoking status. At the time when the samples were collected in 2016, an average of 33.1 % of women aged 25–44 years had a BMI ≥ 25 in Sweden [27], which is a proportion close to the women included in the study, where 30.3% had a BMI ≥ 25. Similarly, in both the study population and the total population in Sweden, an average of 6% reported that they were current smokers [28].

We found that women using PCP more often, in particular perfume, had a higher concentration of the phthalate metabolite MEP in ovarian follicular fluid. MEP is a metabolite of diethyl phthalate (DEP) and one of the most abundant metabolites found in the urine of humans [29]. DEP is the primary phthalate used as a solvent and fixative in fragrances. Our results suggest that perfume use is an important exposure pathway leading all the way to the contamination of the follicular fluid. Phthalates are rapidly metabolised and excreted from the body, and discontinued use of products containing phthalates will decrease exposure. For example, lower levels of phthalate metabolites in ovarian follicular fluid of women seeking assisted reproductive care after the COVID19-lockdown have been suggested to be a result off make-up and fragrance product use during this period [30]. Women are inevitably exposed to phthalates from other sources too. For example, DEP is also widely used as a plasticiser in various plastic products and food-contact materials. Previously, Di Napoli and Yao et al. were able to associate frequent use of shower-gel and consumption of sauces or dressings in plastic containers with the concentration of MEP in urine [31, 32]. Differences in questionnaire design make direct comparisons between studies challenging. For example, our questionnaire covered multiple lifestyle factors but did not specifically collect information on food-packaging or consumption of processed food-products. The questionnaire by Di Napoli et al. on the other hand focused on detailed dietary factors and included dietary supplements and physical activity, but did not cover personal care products and living environment [32]. Animal studies suggest that DEP has potential effects on male reproduction with weaker evidence for female reproductive toxicity [33, 34]. In our previous studies, we did not find an association between MEP levels in follicular fluid and ovarian function [5]. Nonetheless, there are studies that have reported indications that DEP might be associated with higher odds of preterm birth, early onset of puberty or pregnancy loss in humans [34]. Although the outcomes overlap with the observations from experimental animal studies [33], inconsistencies remain regarding the possible effect of DEP on female reproduction [33, 34].

We found that cxMINCH, a metabolite of the phthalate substitute DiNCH, and ∑DiNP were associated with fatty fish intake, although the correlation lost statistical significance after correction for multiple testing. Dietary intake is the predominant route of exposure to DiNCH, whereas for DiNP, both dietary intake and dust ingestion contribute [35]. Data on DiNCH and DiNP in food products are limited, but a study from Canada detected levels in both packaged and non-packaged fish from Canada and South Africa, suggesting that packaging is not the predominant source of DiNCH and DiNP from fish-consumption. However, the same study detected significantly higher levels of DiNCH from butter compared to fish and DiNP was detected in similar concentrations in different food categories [36]. In our study, cxMINCH and DiNP only correlated with intake of fish and fatty fish, and no association between phthalate exposure and for example the use of plastics for microwaving, PVC flooring or other suspected sources related to plastics were found. It remains to be studied if cxMiNCH and DiNP end up in fatty fish during processing, if it is present in the fish itself or which other dietary sources could act as the most important determinants of exposure. PVC flooring in kitchen and bedroom has previously been associated with a higher urine concentration of certain phthalate metabolites [37]. Even though flooring might contribute to exposure, our results suggest other routes of exposure to be of greater importance.

Methyl paraben levels in ovarian follicular fluid were significantly associated with hen’s egg consumption, but not with products usually associated with exposure such as PCPs or pharmaceuticals [38]. Even though the link between egg consumption and exposure is similar to what has previously been reported in adolescents [39], the correlation did not persist after correction of *p* values and needs to be further explored.

We also discovered multiple associations between dietary habits and concentrations of the persistent PFAS chemicals in ovarian follicular fluid. PFASs were in general associated with fish and/or hens’ egg consumption. In particular, PFDA and PFUnDA showed significant correlation with fish consumption and PFOS and PFNA showed significant correlation before correction of *p* values. This is in line with a previous Swedish food basket study, where fish was a major contributor to the dietary exposure to PFNA, PFUnDA, PFDA and PFOS, while not as important pathway for example for PFOA and PFHxS [19]. For hen’s egg consumption, the strongest correlation was seen for PFOS and PFUnDA, while PFNA, PFHxS and PFDA were significant before correction of *p* values. Fish, hens’ eggs and egg products as well as fruit and fruit products are healthy food categories that have previously been considered important contributors to dietary PFAS exposure also on a European level [40]. In Sweden, egg and meat consumption have historically been important sources of dietary intake of PFASs, although with decreasing levels during recent decades [19]. A more recent study found that higher levels of both PFOS and PFUnDA were associated with both healthy eating score and higher diversity in the diet of Swedish adolescents [41]. We also found a correlation between PCP-use and PFASs. These correlations did not persist after correction of *p* values, but the observation could be explained by PFAS used in cosmetics [42]. The evidence of adverse health effects of PFAS exposure related to reproductive outcomes in women is insufficient for drawing firm conclusions [40]. PFASs have been associated with outcomes related to ovarian function in experimental animals and humans [43], but there are also studies showing no effects related to exposure [40]. Our studies in the same cohort of women suggest that PFAS exposure increases ovarian sensitivity to hormone stimulation while reducing embryo quality [6]. In vitro, PFASs show potential to disrupt oocyte- or early embryo development [44–48].

Altogether, the situation where healthy diet is a source of toxic chemicals is complex and the risk-benefit balance is challenging to assess. In Sweden, the National Food Authority recommends reduced intake of certain fish for children and women of reproductive age [49]. These recommendations were originally formed to limit exposure to mercury, PCBs and dioxins, but was recently updated to include possible PFAS contamination of self-caught fish from lakes in Sweden [49]. The consumption of self-caught fish by participants in our study was limited suggesting fish also bred or caught for commercial food trade could potentially be an important source of exposure. Fish accumulate several environmental contaminants, and several studies have recently shown how overconsumption of fish may counteract some beneficial health effects due to increased exposure to PCBs and/or dioxins [50, 51]. Such studies are not available for complex reproductive health outcomes, and underlining the complexity in health-risk assessment and subsequent recommendations for the general human population.

Our study also identified eggs as a potential source of PFAS, and eggs from organic hens the most important contributor. In Sweden, fish meal is commonly added to the feed of organic free range poultry to meet the hen’s need for essential amino acids. It is therefore plausible that feeding the hens fish meal leads to increased PFAS intake and increased levels of PFAS in the eggs.

Although diet and drinking water are important routes of exposure to PFASs, inhalation of household dust or indirect exposure through indoor air can also contribute [18]. Low frequency of vacuum-cleaning or sweeping the floor was in general associated with higher PFAS-exposure. However, these results were not significant in the models adjusted for co-variates. It is possible, that considering the lower proportion of human exposure through inhalation [17, 18], our study population did not have sufficient statistical power to detect these differences.

Our study has limitations. The cohort was relatively small and certain categories of lifestyle data, such as occupation, could not be analysed with sufficient statistical power. Likewise, the cohort lacks power to detect smaller differences in lifestyle and related exposures. We used a questionnaire developed for this cohort. The questionnaire was loosely based on the SELMA cohort [52] to gather a wide range of lifestyle factors. However, the questionnaire had to be kept short due to time restrictions upon patient recruitment during OPU and was not systematically validated. Another limitation of the study is the possible presence of unmeasured confounders. However, we did adjust for age, BMI and parity and it is unlikely that other major influential factors exist. Our target population consists of women seeking care for assisted reproduction. These women may not represent the general public which may affect the potential for extrapolating the results to the average woman of fertile age [53]. Subsequently, the included women may also practice different lifestyle or dietary habits compared to other subpopulations. However, the exposure profile in our cohort [5, 6], correspond to concentrations measured in in a larger cohort of pregnant women in Sweden [54], implying this bias reasonably is small. Further, even if levels of chemical exposures or lifestyle factors in our study differ slightly from the general population of women of fertile age, it is unlikely that the associations we found have noticeable biases.

The study has several strengths too. To our knowledge, this is the first study linking lifestyle factors in combination with dietary habits to levels of EDCs in the ovarian follicular fluid of women of reproductive age. As exposure to EDCs correlate with reduced fertility, it is of utmost importance to identify what modifiable factors contributing to the exposure of the key reproductive organs and cells, ovaries and oocytes. We also think that the associations we discovered are likely to be underestimated due to the questionnaire that was simple and not validated, thus leading to rather blunt estimates. Finally, our study targets an important population: women at an age where fertility starts declining. The average age of women at first childbirth is constantly increasing, reaching already 30 years in Sweden. Fertility starts declining steeply after the age of 35 due to declining quality and quantity of oocytes [55]. It will be important to develop strategies to safeguard fertility in ageing women, where lifestyle recommendations could play an important role.

## Conclusion

To the best of our knowledge, this is the first study showing how lifestyle factors in combination with dietary habits are reflected in the contamination of the ovary by both persistent and semi-persistent chemicals. Determinants of chemical levels were found to include both frequent use of PCP such as perfume use as well as dietary habits such as consumption of fish and eggs. Subsequently, these data can be used to form recommendations regarding lifestyle or diet to mitigate possible negative health outcomes associated with EDC exposure and to help inform chemical policy decision making. For example, discontinued use of PCPs such as perfumes containing phthalates can reduce exposure. Moreover, the presented data add to the current knowledge of fish consumption being related to exposure of certain chemicals or chemical groups. However, we still lack the data whether excessive consumption of fish – and by extent higher exposure to DiNCH metabolites and certain PFASs – prevail over the well-established health benefits of fish in the diet, especially regarding complex traits as ovarian function. This highlights the need for continuous identification of critical effects of chemicals as a high priority task. Finally, our study can also help to design larger intervention studies to examine possible effects of lifestyle changes on chemical exposure levels to unravel the complex interactions between biological factors, lifestyle and chemical exposures in more detail.

### Supplementary information

Supplementary Information

Reporting Checklist

## Data Availability

The data supporting the findings of this study, including individual health, lifestyle, and chemical concentration data, are not openly available due to sensitivity reasons. However, the data can be made available upon reasonable request to the corresponding author, subject to the establishment of data-sharing agreements. The data are securely stored in controlled access data storage at Karolinska Insitutet, Stockholm, Sweden.
