# Supplementary material for: Associations between lifestyle factors and levels of per- and polyfluoroalkyl substances (PFASs), phthalates and parabens in follicular fluid in women undergoing fertility treatment
Source: J Expo Sci Environ Epidemiol. 2023 Jul 22;33(5):699–709. doi: 10.1038/s41370-023-00579-1 (PMC10541317; doi:10.1038/s41370-023-00579-1)
Supplement: Supplementary file 1 — Supplementary Information [file 41370_2023_579_MOESM1_ESM.pdf]

SUPPORTING INFORMATION

Associations between lifestyle factors and levels of per- and polyfluoroalkyl substances (PFASs), phthalates and parabens in follicular fluid in women undergoing fertility treatment

Corresponding author: Ida Hallberg, ida.hallberg@slu.se, +4618-671641

Contents

Figure S1. Correlation between chemicals 2

Figure S2. Directed acyclic graph (DAG) diagram describing the potential effect of measured covariates on the association between lifestyle factors and chemical exposure........ 2

Table S1. Self-administered questionnaire filled in by participating women 3

Table S2. Performance characteristics, chemicals and internal standards used to quantify bisphenols, phthalate metabolites and parabens 5

Table S3: Performance characteristics, chemicals and internal standards used to quantify per- and polyfluoroalkyl substances (PFASs)........ 6

Table S4. Distribution and chemical concentration in ovarian follicular fluid (ng/mL), originally published by Bellavia et al., 2022.[2] 7

Table S5. Proportion of patients above level of detection for the analysed compounds in ovarian follicular fluid 8

Table S6. Quantification of samples in sample blanks 9

Table S7. Spearman's correlation between lifestyle factors and age, BMI and parity........ 9

Table S8. Spearman's correlation between lifestyle factors and chemical levels in follicular fluid, unadjusted for covariates (BMI, Age, Parity) 9

References........13

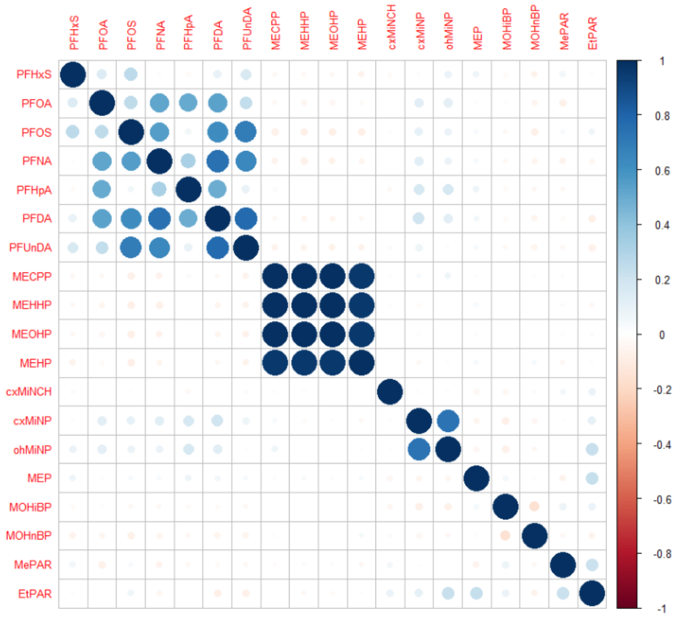

Figure S1. Correlation between chemicals

Pearsons' correlation vizualised by a correlation plot.[1] Area and color intensity of the glyph is proportional to the correlation coefficient and displayed on the right side of the figure. Red represent a negative correlation while blue represent a positive correlation.

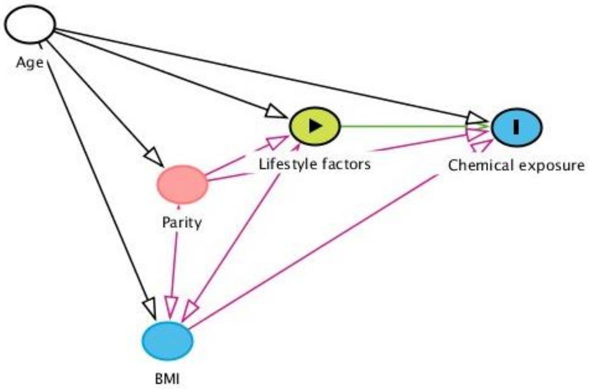

Figure S2. Directed acyclic graph (DAG) diagram describing the potential effect of measured covariates on the association between lifestyle factors and chemical exposure

Age and parity are potential confounders of the association while BMI may act as a potential confounder or be influenced by lifestyle factors and by this be on a causal pathway to chemical exposure.

Table S1. Self-administered questionnaire filled in by participating women

Questionnaire for women undergoing IVF treatment
Environmental impact of female fertility

1. Personal data and home environment

1.1 For how long have you lived in Sweden?

☐ All my life ☐ More than 5 years
☐ Less than 5 years ☐ Not currently

1.2 How often do you vacuum-clean or sweep the floor in your home?

☐ Every day ☐ Once/week
☐ 2-3 times/week ☐ Every other week
☐ Once/month ☐ Less than once/month

1.3 What type of flooring do you have in your home?

	Less then 30% of area	30-60 % of area	More than 60 % of area
Linoleum	☐	☐	☐
Vinyl/PVC	☐	☐	☐
Parquet floor/Hardwood	☐	☐	☐
Floating wood-tile	☐	☐	☐
Carpet	☐	☐	☐
Tiles/Stone	☐	☐	☐
Cork	☐	☐	☐
Other/Unkown	☐	☐	☐

1.4 Do you have own well for water-supply?

☐ Yes ☐ No

1.5 Do you use odour suppressants in your home (sprays, aerosols)?

☐ Yes ☐ No

2. Occupation

2.1 What have your main occupation been the last 6 months?

☐ Student ☐ Unemployed
☐ Sick leave ☐ Domestic work

☐ Work, occupation: ______________________

☐ Other: ________________________________

3. Living environment and health

3.1 Have you used sunscreen the last 6 months?

☐ Yes ☐ No

If yes, how often did you use sunscreen:

☐ Daily ☐ Few times per week
☐ Less then a few times per week

3.2 Do you use snuff? ☐Yes ☐ No

3.3 Does anyone in your family smoke?

☐ No ☐ Yes, daily ☐ Yes, sometimes

3.4 If anyone smoke, where in your home (multiple choice available)?

☐ Outside (balcony/other)
☐ By kitchen fan
☐ In most rooms

3.5 If you used to smoke earlier, how long ago since you quit?

____

3.6 If you used to smoke, how many cigarettes did you smoke on a daily basis?

☐ Single occations ☐ 0-10 cigarettes/day
☐ 10-20 cigarettes/day ☐ More than1 pk/day

3.7 Have you used any medication without prescription the last week (e.g. ibuprofen, paracetamol, allergy medicine, medication against acid reflux/ gastritis)?

☐ Yes, type; ______________________

____

____

☐ No

3.8 Do you eat dietary supplements (e.g. vitamins, minerals, fatty acids)?

☐ Yes, regularly ☐ Yes, sometimes ☐ No

If yes, what type (Name, Manufacturer):

____

____

3.9 Do you use microwave to heat food in plastic lunch-boxes?

☐ Yes
☐ Yes, but only in plastics intended for this use
☐ No

Continue on the following page →

3.10 How often do you use the following products?

	Daily	Few times/week	Few times/month	Rarely/Never
Make-up	☐	☐	☐	☐
Perfume	☐	☐	☐	☐
Hair-dye	☐	☐	☐	☐
Hairspray	☐	☐	☐	☐
Impregnated shoes/clothes (e.g. Gore-Tex®)	☐	☐	☐	☐

3.11 What does your daily diet consist of (multiple choice)?

	How often do You consume the the following products?					Do You usually choose organic alternative?	
	Daily	Every week	Every month	Seldom	Never	Yes	No
Meat	☐	☐	☐	☐	☐	☐	☐
Dairy products	☐	☐	☐	☐	☐	☐	☐
Egg	☐	☐	☐	☐	☐	☐	☐
Fish							
- Fat fisk (e.g. salmon)	☐	☐	☐	☐	☐	☐	☐
- White fish	☐	☐	☐	☐	☐	☐	☐
- Self-caught fish	☐	☐	☐	☐	☐		
Hunted game	☐	☐	☐	☐	☐		
Bread, grain-products	☐	☐	☐	☐	☐	☐	☐
Soy products	☐	☐	☐	☐	☐	☐	☐
Vegetables, fruit	☐	☐	☐	☐	☐	☐	☐
Coffee	☐	☐	☐	☐	☐	☐	☐
Fried food, chips	☐	☐	☐	☐	☐		
Barbeque, smoked food	☐	☐	☐	☐	☐		
Alcohol	☐	☐	☐	☐	☐		

4. Other:

4.1 May we contact you to ask complementary questions at a later time? ☐ Yes ☐ No

THANK YOU FOR YOUR PARTICIPATION!

Table S2. Performance characteristics, chemicals and internal standards used to quantify bisphenols, phthalate metabolites and parabens

Compound	Cas-nb	Internal standard
Mono(2-ethyl-5-carboxypentyl) phthalate (MECPP)	40809-41-4	MECPP-13C4 (ring-1,2 & dicarboxyl)
Mono(2-ethyl-5-hydroxyhexyl) phthalate (MEHHP)	40321-99-1	MEHHP-13C4 (ring-1,2 & dicarboxyl)
Mono(2-ethyl-5-oxohexyl) phthalate (MEOHP)	40321-98-0	MEOHP-13C4 (ring-1,2 & dicarboxyl)
Mono(2-ethyl-1-hexyl) phthalate (MEHP)	4376-20-9	MEHP-13C4 (ring-1,2 & dicarboxyl)
Mono-n-octyl phthalate (MnOP)	5393-19-1	MnOP-13C4 (ring-1,2 & dicarboxyl)
Cyclohexane-1,2-dicarboxylic acid, mono-(7-carboxy-4-methylheptyl ester) (cxMiNCH)	1637562-51-6	cxMiNCH-13C4 (ring-1,2 & dicarboxyl)
Cyclohexane-1,2-dicaboxylic acid mono-(7-hydroxy-4-methyloctyl)ester (ohMiNCH)	1637562-52-7	ohMiNCH-13C4 (ring-1,2 & dicarboxyl)
Cyclohexane-1,2-dicaboxylic acid mono-(4-methyl-7-oxooctyl)ester (oxoMiNCH)	1588520-62-0	oxoMiNCH-13C4 (ring-1,2 & dicarboxyl)
Mono-(4-methyl-7-carboxy heptyl)phthalate (cxMiNP)	936022-02-5	cxMiNP-13C4 (ring-1,2 & dicarboxyl)
Mono-(4-methyl-7-hydroxy octyl)phthalate (ohMiNP)	936021-98-6	ohMiNP-13C4 (ring-1,2 & dicarboxyl)
Monoethyl phthalate (MEP)	2306-33-4	MEP-13C4 (ring-1,2 & dicarboxyl)
Monocyclohexyl phthalate (MCHP)	7517-36-4	MCHP-13C4 (ring-1,2 & dicarboxyl)
Monoisobutyl phthalate (MiBP)	30833-53-5	MiBP-13C4 (ring-1,2 & dicarboxyl)
Mono-n-butyl phthalate (MnBP)	131-70-4	MnBP-13C4 (ring-1,2 & dicarboxyl)
Mono(2-hydroxyisobutyl)phthalate (MOHiBP)	64339-39-5	MOHiBP-d4-3,4,5,6
Mono(3-hydroxybutyl)phthalate (MOHBP)	57074-43-8	MOHBP-d4-3,4,5,6
Methyl paraben (MePAR)	99-76-3	MePAR-d4-2,3,5,6
Ethyl paraben (EtPAR)	120-47-8	EtPAR-d4-2,3,5,6
Isopropyl paraben (iPrPAR)	4191-73-5	iPrPAR-d4-2,3,5,6
Propyl paraben (PrPAR)	94-13-3	PrPAR-d4-2,3,5,6
Isobutyl paraben (iBuPAR)	4247-02-3	iBuPAR-d4-2,3,5,6
Butyl paraben (BuPAR)	94-26-8	BuPAR-d4-2,3,5,6
Bisphenol A (BPA)	80-05-7	BPA-d18
2,4-Bis(4-hydroxyphenyl)-4- methyl-1-pentene (MBP)	13464-24-9	MBP-d18
Bisphenol F (BPF)	620-92-8	BPF-13C12 (phenoxy)
Bisphenol S (BPS)	80-09-1	BPS-13C12 (phenoxy)
4-Hydroxyphenyl 4-Isopropoxyphenyl Sulfone (BSIP)	95235-30-6	BPSIP d7 (butyl)
Bisphenol AF (BPAF)	1478-61-1	Bisphenol-AF d4 - 6,6',8,8'
Bisphenol AP (BPAP)	1571-75-1	BPAP-d5 (phenyl)
Bisphenol B (BPB)	77-40-7	Bisphenol-B (phenoxy)d8
Bisphenol E (BPE)	2081-08-5	BPE-13C6 (phenoxy)
Bisphenol Z (BPZ)	843-55-0	Bisphenol-Z (phenoxy)13C12

The native and isotopically labeled compounds were purchased from Merck, Toronto Research Chemicals, Cambridge Isotope laboratories, Chiron, CDN Isotopes and Wellington Laboratories.

Performance characteristics bisphenols, phthalate metabolites and parabens[2]

The recovery and repeatability of the analytical method were determined using fortified bovine follicular fluid at a level of 2 ng/ml (n=8). In parallel with the samples (n = 333) 22 blanks (MQ) were analyzed. The collected blank data were used for the determination of the limits of detection (LODs) and limits of quantification (LOQs). The LOD was defined as three times the standard deviation of the blank plus the average blank value. The LOQ was defined as 3.3 * LOD value. The fortified bovine follicular fluid samples were also analyzed as quality control sample included in each LC-MS/MS run. The reproducibility was determined with the values of the quality control samples (n=8 in the course of the analyses).

Table S3: Performance characteristics, chemicals and internal standards used to quantify per- and polyfluoroalkyl substances (PFASs)

Compound	Cas no	Internal standard
Perfluoro-1-hexanesulfonate (PFHxS)	355-46-4	PFHxS-13C3 (1,2,3)
Perfluoro-n-heptanoic acid (PFHpA)	375-85-9	PFHpA-13C4 (1,2,3,4)
Perfluoro-n-octanoic acid (PFOA)	335-67-1	PFOA-13C8
Perfluoro-n-nonanoic acid (PFNA)	375-95-1	PFNA-13C9
Perfluoro-n-decanoic acid (PFDA)	335-76-2	PFDA-13C6 (1,2,3,4,5,6)
Perfluoro-n-undecanoic acid (PFUnDA)	2058-94-8	PFUnDA-13C7 (1,2,3,4,5,6,7)
Perfluoro-n-dodecanoic acid (PFDoDA)	307-55-1	PFDoDA-13C2 (1,2)
Perfluoro-octanesulfonate (PFOS)	1763-23-1	PFOS-13C8

The native and isotopically labeled compounds were purchased from Merck, Toronto Research Chemicals, Cambridge Isotope laboratories, Chiron, CDN Isotopes and Wellington Laboratories.

Performance characteristics PFAS[3]

LOD and LOQ were defined as the concentration corresponding to 3 and 10 times the standard deviation of the ratio of the peak at the same retention time as the analyzed compounds and the corresponding internal standard divided by the slope of the calibration line and determined in the chemical blank samples

Table S4. Distribution and chemical concentration in ovarian follicular fluid (ng/mL), originally published by Bellavia et al., 2022.[2]

	Mean (SD)	Median [min, max]	Non-quantified, n (%)
**MEHP**	1.04 (1.65)	0.78 [0.48, 21.00]	25 (13.5%)
**MECPP**	0.39 (1.85)	0.19 [0.06, 25.00]	3 (1.6%)
**MEHHP**	0.08 (0.35)	0.04 [0.02, 4.70]	5 (2.7%)
**MEOHP**	0.05 (0.25)	0.02 [0.01, 3.30]	10 (5.4%)
**ΣDEHP**[a]	0.004 (0.002)	0.004 [0.0003, 0.014]	4 (2.2%)
**MEP**	0.98 (1.10)	0.74 [0.29, 10.00]	4 (2.2%)
**cxMiNP**	2.52 (7.03)	0.61 [0.10, 70.00]	3 (1.6%)
**MOHiBP**	0.07 (0.05)	0.05 [0.02, 0.41]	13 (7.0%)
**Methylparaben**	152.69 (653.76)	7.9 [0.13, 6000.00]	5 (2.7%)
**PFHxS**	1.15 (1.69)	0.60 [0.01, 13.00]	5 (2.7%)
**PFOA**	1.35 (0.90)	1.16 [0.17, 8.05]	4 (2.2%)
**PFOS**	4.09 (2.43)	3.54 [0.17, 15.05]	4 (2.2%)
**PFNA**	0.58 (0.35)	0.51 [0.02, 3.16]	4 (2.2%)
**PFUnDA**	0.23 (0.13)	0.21 [0.04, 0.68]	13 (7%)
**PFDA**	0.28 (0.15)	0.26 [0.06, 1.09]	5 (2.7%)

[a]Molecular sum of MEHP, MECPP, MEHHP, MEOHP with the unit of mol/mL.

Table S5. Proportion of patients above level of detection for the analysed compounds in ovarian follicular fluid

Name	n	LOD	> LOD	Mean	Median	Min	Max
Perfluoro-n-octanoic acid	181	0.06	1.00	1.35	1.161	0.17	8.05
Perfluoro-octanesulfonate (sum)	181	0.04	1.00	4.09	3.54	0.17	15.05
Perfluoro-n-nonanoic acid	181	0.01	1.00	0.58	0.50	0.02	3.16
Mono-(2-ethyl-5-carboxypentyl) phthalate	183	0.02	1.00	0.39	0.19	0.01	25
Mono-(4-methyl-7-carboxyheptyl)phthalate	183	0.02	1.00	2.50	0.61	0.01	70
Monoethyl phthalate	183	0.21	1.00	0.97	0.74	0.11	10
Methyl paraben	183	0.10	1.00	255	8	0.10	8800
Mono-(2-ethyl-5-hydroxyhexyl) phthalate	183	0.02	0.99	0.07	0.04	0.01	4.7
Perfluoro-1-hexanesulfonate	181	0.01	0.99	1.14	0.59	0.01	13.0
Perfluoro-n-decanoic acid	181	0.02	0.99	0.28	0.25	0.01	1.09
Mono-(4-methyl-7-hydroxyoctyl)phthalate	183	0.01	0.98	0.99	0.26	0.01	33
Cyclohexane-1,2-dicarboxyl acid, mono-(7-carboxy-4-methylheptyl ester)	183	0.01	0.98	0.42	0.06	0.01	16
Mono(2-ethyl-5-oxohexyl) phthalate	183	0.01	0.96	0.05	0.02	0.01	3.3
Mono(2-hydroxyisobutyl)phthalate	183	0.02	0.95	0.06	0.05	0.01	0.41
Perfluoro-n-undecanoic acid	181	0.04	0.95	0.22	0.20	0.02	0.68
Mono-(2-ethyl-1-hexyl) phthalate	183	0.53	0.90	0.95	0.73	0.27	21
Perfluoro-n-heptanoic acid	181	0.01	0.80	0.03	0.02	0.01	0.49
Mono(3-hydroxybutyl)phthalate	183	0.03	0.65	0.84	0.03	0.02	42
Ethyl paraben	183	0.03	0.50	0.14	0.02	0.02	2.3
Propyl paraben	183	0.08	0.41	0.26	0.04	0.04	11
Cyclohexane-1,2-dicaboxylic acid mono-(7-hydroxy-4- methyloctyl)ester	183	0.03	0.38	0.14	0.02	0.02	8.6
Cyclohexane-1,2-dicaboxylic acid mono-(4-methyl-7-oxooctyl)ester	183	0.03	0.38	0.04	0.02	0.02	1.5
Isopropyl paraben	183	0.01	0.32	0.05	0.03	0.01	0.46
Butyl paraben	183	0.05	0.28	0.16	0.03	0.03	9.4
Monoisobutyl phthalate	183	2.48	0.26	1.72	1.24	1.24	7
Bisphenol S	183	0.06	0.17	0.04	0.03	0.03	0.11
Isobutyl paraben	183	0.01	0.14	0.01	0.01	0.01	0.16
Monocyclohexyl phthalate	183	0.01	0.11	0.01	0.01	0.01	0.18
4-Hydroxyphenyl 4-Isopropoxyphenyl Sulfone	183	0.04	0.11	0.03	0.02	0.02	0.47
Bisphenol F	183	0.03	0.09	0.02	0.02	0.02	0.28
Mono-n-butyl phthalate	183	3.06	0.09	1.73	1.53	1.53	5.2
Bisphenol A	183	1.04	0.06	0.59	0.52	0.52	2.3
Mono-n-octyl phthalate	183	0.01	0.03	0.01	0.01	0.01	0.02
2,4-Bis(4-hydroxyphenyl)-4- methyl-1-pentene	183	0.05	0.03	0.03	0.03	0.03	0.14
Perfluoro-n-dodecanoic acid	181	0.05	0.02	0.03	0.03	0.03	0.09
Bisphenol AF	183	0.02	0.01	0.01	0.01	0.01	0.17
Bisphenol B	183	0.02	0.01	0.01	0.01	0.01	0.01
Bisphenol AP	183	0.04	0.00	0.02	0.02	0.02	0.02
Bisphenol E	183	0.05	0.00	0.03	0.03	0.03	0.03
Bisphenol Z	183	0.10	0.00	0.05	0.05	0.05	0.05

Table S6. Quantification of chemicals in sample blanks

Compound	>LOD in blanks	Mean (detected)	LOD
MECPP	0/6	0.006	0.02
MEHHP	0/6	0.005	0.02
MEOHP*	6/6	0.012	0.01
MEHP	0/6	0.203	0.53
MEP	0/6	0.070	0.21
MOHiBP	0/6	0.005	0.02
MOHnBP	0/6	0.009	0.03
MePAR	0/6	0.065	0.10
EtPAR	0/6	0.018	0.03
PrPAR	0/6	0.03	0.08
BPS**	1/6	0.49	0.06
MEHP	0/4	0.110	0.53
MOHiBP	0/4	0.005	0.02
MePAR	0/4	0.025	0.10
EtPAT	0/4	0.018	0.03
iPrPAR	0/4	0.001	0.01
PrPAR	0/4	0.02	0.08
BPSIP**	1/4	0.01	0.04

*MEOHP was quantified in sample blanks in concentrations of approximately 20% of the levels in ovarian follicular fluid. The correlation with the other metabolites of DEHP remained high and sensitivity-analysis excluding MEOHP from DEHP-metabolites generated similar results.
**BPS was quantified in one sample blank and BPSIP in rinse media, but these analytes were not included in the analyses as the detection levels in women were <20%.

Table S7. Spearman's correlation between lifestyle factors and age, BMI and parity

Variable	Age	BMI	Parity
**Cleaning**	0.13, p =0.07	0.13, p=0.09	-0.04, p=0.54
**White fish**	-0.13, p=0.08	0.21, p=0.005	-0.19, p=0.01
**Egg**	0.03 p = 0.64	0.03, p =.65	0.09, p=0.21
**Microwave**	-0.10, p=0.17	0.003, p=0.96	0.01, p=0.85
**Meat**	0.01, p=0.83	0.04, p=0.54	-0.08, p=0.23
**Fatty fish**	-0.10, p=0.15	0.16, p=0.02	-0.09, p=0.19
**Fish mean**	-0.13, p=0.07	0.20, p=0.004	-0.15, p=0.03
**Make-up**	-0.03, p=0.60	0.11, p=0.11	-0.17, p=0.02
**Fragrance**	0.10, p=0.15	-0.14, p=0.04	0.01, p=0.86
**Hairspray**	-0.04, p=0.58	-0.03, p=0.62	-0.12, p=0.09
**Impregnated clothes**	-0.05, p=0.45	0.12, p=0.09	0.02, p=0.70

Table S8. Spearman's correlation between lifestyle factors and chemical levels in follicular fluid, unadjusted for covariates (BMI, Age, Parity)

Variable 1	Variable 2	N	r(95% CI) unadjusted	p-value	p-corr[a]
PFHxS	PCP_makeup	179	0.03 (-0.11 ; 0.18)	0.6678	.
PFHxS	PCP_perfume	177	0.07 (-0.08 ; 0.22)	0.3336	.
PFHxS	PCP_hairspray	169	0.02 (-0.14 ; 0.17)	0.8369	.
**PFHxS**	**PCP_impreshoes**	**171**	**0.18 (0.03 ; 0.32)**	**0.0157**	.
PFHxS	sum_PCP	180	0.03 (-0.12 ; 0.17)	0.7372	.
PFHxS	flooring_gr	181	0.03 (-0.11 ; 0.18)	0.6499	.
**PFHxS**	**Cleaning**	**177**	**0.22 (0.07 ; 0.36)**	**0.0032**	.
PFHxS	Microwave_use	181	0.04 (-0.10 ; 0.19)	0.5583	.
**PFHxS**	**Diet_meat**	**179**	**0.19 (0.04 ; 0.32)**	**0.0122**	.
PFHxS	mean_Diet_fish	180	0.04 (-0.11 ; 0.18)	0.6022	.
PFHxS	Diet_fattyfish	175	0.04 (-0.11 ; 0.18)	0.6203	.
PFHxS	Diet_whitefish	173	0.06 (-0.09 ; 0.21)	0.4258	.
PFOS	PCP_makeup	179	0.06 (-0.09 ; 0.20)	0.4527	.
PFOS	PCP_perfume	177	0.03 (-0.12 ; 0.18)	0.6809	.
PFOS	PCP_hairspray	169	0.10 (-0.05 ; 0.24)	0.2089	.
PFOS	PCP_impreshoes	171	0.08 (-0.07 ; 0.23)	0.3032	.
PFOS	sum_PCP	180	0.03 (-0.12 ; 0.17)	0.7289	.

PFOS	flooring gr	181	0.08 (-0.07 ; 0.22)	0.3116	.
**PFOS**	**Cleaning**	**177**	**0.27 (0.12 ; 0.40)**	**0.0003**	**0.0308**
**PFOS**	**Microwave use**	**181**	**0.17 (0.02 ; 0.30)**	**0.0253**	**.**
PFOS	Diet meat	179	0.03 (-0.12 ; 0.17)	0.7385	.
PFOS	mean Diet fish	180	0.11 (-0.04 ; 0.25)	0.1435	.
PFOS	Diet fattyfish	175	0.10 (-0.04 ; 0.25)	0.1666	.
**PFOS**	**Diet whitefish**	**173**	**0.22 (0.07 ; 0.35)**	**0.0041**	**.**
**PFOS**	**Diet egg**	**176**	**0.27 (0.13 ; 0.41)**	**0.0002**	**0.0201**
PFNA	PCP makeup	179	0.13 (-0.02 ; 0.27)	0.0929	.
PFNA	PCP perfume	177	0.01 (-0.14 ; 0.16)	0.8907	.
PFNA	PCP hairspray	169	0.15 (-0.01 ; 0.29)	0.0590	.
PFNA	PCP impreshoes	171	0.08 (-0.07 ; 0.22)	0.3186	.
PFNA	sum PCP	180	0.11 (-0.04 ; 0.25)	0.1523	.
PFNA	flooring gr	181	0.12 (-0.03 ; 0.26)	0.1166	.
**PFNA**	**Cleaning**	**177**	**0.16 (0.02 ; 0.30)**	**0.0296**	**.**
**PFNA**	**Microwave use**	**181**	**0.16 (0.02 ; 0.30)**	**0.0281**	**.**
PFNA	Diet meat	179	0.10 (-0.04 ; 0.25)	0.1649	.
**PFNA**	**mean Diet fish**	**180**	**0.17 (0.02 ; 0.31)**	**0.0216**	**.**
**PFNA**	**Diet fattyfish**	**175**	**0.17 (0.03 ; 0.31)**	**0.0215**	**.**
**PFNA**	**Diet whitefish**	**173**	**0.27 (0.12 ; 0.40)**	**0.0003**	**0.0338**
**PFNA**	**Diet egg**	**176**	**0.22 (0.08 ; 0.36)**	**0.0030**	**.**
PFDA	PCP makeup	179	0.14 (-0.01 ; 0.28)	0.0681	.
PFDA	PCP perfume	177	0.05 (-0.09 ; 0.20)	0.4750	.
**PFDA**	**PCP hairspray**	**169**	**0.19 (0.04 ; 0.33)**	**0.0129**	**.**
PFDA	PCP impreshoes	171	0.08 (-0.07 ; 0.23)	0.2788	.
PFDA	sum PCP	180	0.10 (-0.05 ; 0.24)	0.2027	.
PFDA	flooring gr	181	0.10 (-0.05 ; 0.24)	0.1945	.
PFDA	Cleaning	177	0.12 (-0.02 ; 0.27)	0.1000	.
PFDA	Microwave use	181	0.14 (-0.01 ; 0.28)	0.0630	.
PFDA	Diet meat	179	0.01 (-0.13 ; 0.16)	0.8589	.
PFDA	mean Diet fish	180	0.14 (-0.00 ; 0.28)	0.0541	.
PFDA	Diet fattyfish	175	0.11 (-0.04 ; 0.25)	0.1533	.
**PFDA**	**Diet whitefish**	**173**	**0.30 (0.16 ; 0.43)**	**<.0001**	**0.0054**
**PFDA**	**Diet egg**	**176**	**0.24 (0.09 ; 0.37)**	**0.0013**	**.**
PFUnDA	PCP makeup	179	0.11 (-0.04 ; 0.25)	0.1438	.
PFUnDA	PCP perfume	177	0.11 (-0.04 ; 0.25)	0.1554	.
**PFUnDA**	**PCP hairspray**	**169**	**0.17 (0.02 ; 0.31)**	**0.0262**	**.**
PFUnDA	PCP impreshoes	171	0.13 (-0.02 ; 0.27)	0.1002	.
PFUnDA	sum PCP	180	0.06 (-0.09 ; 0.20)	0.4249	.
**PFUnDA**	**flooring gr**	**181**	**0.15 (0.01 ; 0.29)**	**0.0381**	**.**
**PFUnDA**	**Cleaning**	**177**	**0.18 (0.03 ; 0.32)**	**0.0183**	**.**
**PFUnDA**	**Microwave use**	**181**	**0.19 (0.05 ; 0.33)**	**0.0087**	**.**
PFUnDA	Diet meat	179	0.05 (-0.10 ; 0.20)	0.4949	.
**PFUnDA**	**mean Diet fish**	**180**	**0.20 (0.06 ; 0.34)**	**0.0057**	**.**
PFUnDA	Diet fattyfish	175	0.14 (-0.00 ; 0.29)	0.0550	.
**PFUnDA**	**Diet whitefish**	**173**	**0.37 (0.23 ; 0.49)**	**<.0001**	**<.0001**
**PFUnDA**	**Diet egg**	**176**	**0.24 (0.09 ; 0.37)**	**0.0013**	**.**
sumDEHP	PCP makeup	181	0.06 (-0.08 ; 0.21)	0.3961	.
**sumDEHP**	**PCP perfume**	**179**	**0.20 (0.05 ; 0.33)**	**0.0080**	**.**
sumDEHP	PCP hairspray	171	0.05 (-0.10 ; 0.20)	0.4914	.
sumDEHP	PCP impreshoes	172	0.03 (-0.12 ; 0.18)	0.7117	.
sumDEHP	sum PCP	182	0.07 (-0.07 ; 0.22)	0.3239	.
sumDEHP	flooring gr	183	0.08 (-0.07 ; 0.22)	0.2898	.
sumDEHP	Cleaning	179	0.14 (-0.01 ; 0.28)	0.0598	.
sumDEHP	Microwave use	183	0.07 (-0.08 ; 0.21)	0.3618	.
sumDEHP	Diet meat	181	0.12 (-0.02 ; 0.26)	0.0962	.

sumDEHP	mean Diet fish	182	0.00 (-0.14 ; 0.15)	0.9616	.
sumDEHP	Diet fattyfish	177	0.03 (-0.12 ; 0.17)	0.7159	.
sumDEHP	Diet whitefish	174	0.00 (-0.14 ; 0.15)	0.9480	.
sumDEHP	Diet egg	178	0.07 (-0.08 ; 0.22)	0.3456	.
sumDEHP MEOHP	PCP makeup	181	0.06 (-0.08 ; 0.21)	0.3841	.
**sumDEHP MEOHP**	**PCP perfume**	**179**	**0.20 (0.05 ; 0.33)**	**0.0076**	**.**
sumDEHP MEOHP	PCP hairspray	171	0.05 (-0.10 ; 0.20)	0.5060	.
sumDEHP MEOHP	PCP impreshoes	172	0.03 (-0.12 ; 0.18)	0.7083	.
sumDEHP MEOHP	sum PCP	182	0.07 (-0.07 ; 0.22)	0.3302	.
sumDEHP MEOHP	flooring gr	183	0.08 (-0.07 ; 0.22)	0.2884	.
sumDEHP MEOHP	Cleaning	179	0.14 (-0.01 ; 0.28)	0.0624	.
sumDEHP MEOHP	Microwave use	183	0.07 (-0.08 ; 0.21)	0.3418	.
sumDEHP MEOHP	Diet meat	181	0.12 (-0.02 ; 0.27)	0.0942	.
sumDEHP MEOHP	mean Diet fish	182	0.00 (-0.14 ; 0.15)	0.9564	.
sumDEHP MEOHP	Diet fattyfish	177	0.03 (-0.12 ; 0.17)	0.7214	.
sumDEHP MEOHP	Diet whitefish	174	0.00 (-0.14 ; 0.15)	0.9559	.
sumDEHP MEOHP	Diet egg	178	0.07 (-0.08 ; 0.21)	0.3592	.
sumDiNP	PCP makeup	181	0.11 (-0.04 ; 0.25)	0.1392	.
sumDiNP	PCP perfume	179	0.00 (-0.14 ; 0.15)	0.9573	.
sumDiNP	PCP hairspray	171	0.03 (-0.12 ; 0.18)	0.6546	.
sumDiNP	PCP impreshoes	172	0.00 (-0.15 ; 0.15)	0.9818	.
sumDiNP	sum PCP	182	0.07 (-0.07 ; 0.22)	0.3223	.
sumDiNP	flooring gr	183	0.05 (-0.09 ; 0.20)	0.4652	.
sumDiNP	Cleaning	179	0.05 (-0.10 ; 0.19)	0.5274	.
sumDiNP	Microwave use	183	0.06 (-0.09 ; 0.20)	0.4498	.
sumDiNP	two Diet meat	181	0.09 (-0.05 ; 0.24)	0.2118	.
**sumDiNP**	**Diet meat**	**181**	**0.16 (0.01 ; 0.30)**	**0.0355**	**.**
**sumDiNP**	**mean Diet fish**	**182**	**0.17 (0.02 ; 0.31)**	**0.0228**	**.**
**sumDiNP**	**Diet fattyfish**	**177**	**0.19 (0.04 ; 0.33)**	**0.0105**	**.**
sumDiNP	Diet whitefish	174	0.13 (-0.02 ; 0.27)	0.0901	.
sumDiNP	Diet egg	178	0.02 (-0.13 ; 0.16)	0.8100	.
cxMiNCH	PCP makeup	181	0.06 (-0.09 ; 0.20)	0.4240	.
cxMiNCH	PCP perfume	179	0.10 (-0.04 ; 0.25)	0.1705	.
cxMiNCH	PCP hairspray	171	0.09 (-0.06 ; 0.23)	0.2552	.
cxMiNCH	PCP impreshoes	172	0.01 (-0.14 ; 0.16)	0.8700	.
cxMiNCH	sum PCP	182	0.08 (-0.07 ; 0.22)	0.3011	.
cxMiNCH	flooring gr	183	0.02 (-0.13 ; 0.16)	0.7998	.
cxMiNCH	Cleaning	179	0.08 (-0.06 ; 0.23)	0.2637	.
cxMiNCH	Microwave use	183	0.11 (-0.04 ; 0.25)	0.1356	.
cxMiNCH	Diet meat	181	0.07 (-0.08 ; 0.21)	0.3645	.
**cxMiNCH**	**mean Diet fish**	**182**	**0.20 (0.06 ; 0.34)**	**0.0061**	**.**
**cxMiNCH**	**Diet fattyfish**	**177**	**0.25 (0.11 ; 0.38)**	**0.0007**	**.**
cxMiNCH	Diet whitefish	174	0.12 (-0.03 ; 0.27)	0.1048	.
cxMiNCH	Diet egg	178	0.08 (-0.06 ; 0.23)	0.2609	.
**MEP**	**PCP makeup**	**181**	**0.17 (0.03 ; 0.31)**	**0.0182**	**.**
**MEP**	**PCP perfume**	**179**	**0.35 (0.22 ; 0.47)**	**<.0001**	**0.0001**
MEP	PCP hairspray	171	0.11 (-0.04 ; 0.26)	0.1465	.
MEP	PCP impreshoes	172	0.02 (-0.13 ; 0.17)	0.7571	.
**MEP**	**sum PCP**	**182**	**0.25 (0.11 ; 0.38)**	**0.0006**	**.**
MEP	flooring gr	183	0.05 (-0.10 ; 0.19)	0.5053	.
MEP	Cleaning	179	0.03 (-0.12 ; 0.17)	0.7116	.
MEP	Microwave use	183	0.10 (-0.04 ; 0.24)	0.1634	.
MEP	Diet meat	181	0.01 (-0.14 ; 0.15)	0.9457	.
MEP	mean Diet fish	182	0.12 (-0.02 ; 0.26)	0.1030	.
MEP	Diet fattyfish	177	0.13 (-0.01 ; 0.27)	0.0770	.
MEP	Diet whitefish	174	0.10 (-0.05 ; 0.24)	0.2077	.

MEP	Diet egg	178	0.10 (-0.05 ; 0.24)	0.1965	.
MOHiBP	PCP makeup	181	0.07 (-0.07 ; 0.22)	0.3231	.
MOHiBP	PCP perfume	179	0.05 (-0.10 ; 0.19)	0.5264	.
MOHiBP	PCP hairspray	171	0.10 (-0.05 ; 0.24)	0.2048	.
MOHiBP	PCP impreshoes	172	0.00 (-0.15 ; 0.15)	0.9625	.
MOHiBP	sum PCP	182	0.11 (-0.03 ; 0.25)	0.1326	.
MOHiBP	flooring gr	183	0.07 (-0.08 ; 0.21)	0.3808	.
MOHiBP	Cleaning	179	0.08 (-0.07 ; 0.22)	0.2821	.
MOHiBP	Microwave use	183	0.02 (-0.12 ; 0.17)	0.7575	.
**MOHiBP**	**Diet meat**	**181**	**0.16 (0.01 ; 0.30)**	**0.0333**	.
MOHiBP	mean Diet fish	182	0.01 (-0.13 ; 0.16)	0.8696	.
MOHiBP	Diet fattyfish	177	0.03 (-0.12 ; 0.18)	0.6742	.
MOHiBP	Diet whitefish	174	0.08 (-0.07 ; 0.23)	0.2788	.
**MOHiBP**	**Diet egg**	**178**	**0.15 (0.01 ; 0.29)**	**0.0408**	.
MePAR	PCP makeup	181	0.00 (-0.14 ; 0.15)	0.9581	.
MePAR	PCP perfume	179	0.11 (-0.04 ; 0.25)	0.1509	.
MePAR	PCP hairspray	171	0.06 (-0.09 ; 0.21)	0.4024	.
MePAR	PCP impreshoes	172	0.04 (-0.11 ; 0.19)	0.6295	.
MePAR	sum PCP	182	0.05 (-0.09 ; 0.20)	0.4645	.
MePAR	flooring gr	183	0.01 (-0.13 ; 0.16)	0.8590	.
MePAR	Cleaning	179	0.03 (-0.12 ; 0.17)	0.7305	.
MePAR	Microwave use	183	0.00 (-0.14 ; 0.15)	0.9935	.
MePAR	Diet meat	181	0.06 (-0.09 ; 0.20)	0.4158	.
MePAR	mean Diet fish	182	0.05 (-0.09 ; 0.20)	0.4899	.
MePAR	Diet fattyfish	177	0.07 (-0.08 ; 0.21)	0.3830	.
MePAR	Diet whitefish	174	0.03 (-0.12 ; 0.17)	0.7409	.
**MePAR**	**Diet egg**	**178**	**0.17 (0.03 ; 0.31)**	**0.0191**	.
PFHxS	PVC scale1	181	0.18 (0.03 ; 0.32)	**0.0151**	.
PFOA	PVC scale1	181	0.11 (-0.03 ; 0.25)	0.1315	.
PFOS	PVC scale1	181	0.10 (-0.05 ; 0.24)	0.1920	.
PFNA	PVC scale1	181	0.09 (-0.06 ; 0.23)	0.2441	.
PFDA	PVC scale1	181	0.07 (-0.07 ; 0.22)	0.3193	.
PFUnDA	PVC scale1	181	0.08 (-0.07 ; 0.22)	0.2781	.
sumDEHP	PVC scale1	183	0.16 (0.02 ; 0.30)	**0.0258**	.
sumMiNP	PVC scale1	183	0.03 (-0.11 ; 0.18)	0.6431	.
cxMiNCH	PVC scale1	183	0.08 (-0.07 ; 0.22)	0.3099	.
MEP	PVC scale1	183	0.02 (-0.13 ; 0.16)	0.7920	.
MOHiBP	PVC scale1	183	0.05 (-0.09 ; 0.20)	0.4736	.
MePAR	PVC scale1	183	0.03 (-0.11 ; 0.18)	0.6409	.

[a]corrected for multiple testing using MEFF.[4] sumDEHP was created by summation of all four metabolites of di-2-ethylhexyl phthalate (DEHP) [mono-2-ethylhexyl phthalate (MEHP), mono-(2-ethyl-5-carboxypentyl) phthalate (MECPP), mono-(2-ethyl-5-hydroxyhexyl) phthalate (MEHHP), and mono(2-ethyl-5-oxohexyl) phthalate (MEOHP)] divided by their molecular weight. ∑DiNP was created by summation of the two secondary metabolites mono-(4-methyl-7-hydroxyoctyl)phthalate (cxMiNP) and monoethyl phthalate (ohMiNP) divided by their molecular weight. The variable “Impreshoes” describe the use of impregnating clothing or shoes, estimated by the patients by the questionnaire used in the study. PVC-scale indicate proportion of the flooring of the household containing PVC flooring (none, <30%, 30-60%, >60%).

References

1. Wei T, V S. R package 'corrplot': Visualization of a Correlation Matrix. (Version 0.92). In: https://github.com/taiyun/corrplot., 2021.

2. Bellavia A, Zou R, Bjorvang RD, Roos K, Sjunnesson Y, Hallberg I *et al* Association between chemical mixtures and female fertility in women undergoing assisted reproduction in Sweden and Estonia. Environmental research 2022; e-pub ahead of print 2022/10/02; doi 10.1016/j.envres.2022.114447. 114447.

3. Bjorvang RD, Hallberg I, Pikki A, Berglund L, Pedrelli M, Kiviranta H *et al* Follicular fluid and blood levels of persistent organic pollutants and reproductive outcomes among women undergoing assisted reproductive technologies. Environmental research 2022; e-pub ahead of print 2022/01/02; doi 10.1016/j.envres.2021.112626. 112626.

4. Li J, Ji L Adjusting multiple testing in multilocus analyses using the eigenvalues of a correlation matrix. Heredity (Edinb) 2005; 95: 221-227.
